# A Case of Systemic Infection Caused by *Streptococcus pyogenes* Oral Infection in an Edentulous Patient

**DOI:** 10.3390/diseases5030017

**Published:** 2017-08-18

**Authors:** Yumi Inagaki, Masanobu Abe, Ryoko Inaki, Liang Zong, Hideyuki Suenaga, Takahiro Abe, Kazuto Hoshi

**Affiliations:** 1 Department of Oral & Maxillofacial Surgery, University of Tokyo Hospital, 7-3-1 Hongo, Bunkyo-ku, Tokyo 113-8655, Japan; 17yumiii@gmail.com (Y.I.); rinaki-tky@umin.net (R.I.); SUENAGAH-ORA@h.u-tokyo.ac.jp (H.S.); ABET-ORA@h.u-tokyo.ac.jp (T.A.); HOSHI-ORT@h.u-tokyo.ac.jp (K.H.); 2 Division for Health Service Promotion, University of Tokyo, 7-3-1 Hongo, Bunkyo-ku, Tokyo 113-003, Japan; 3 Key Laboratory of Carcinogenesis and Translational Research (Ministry of Education), Department of Gastrointestinal Surgery, Peking University Cancer Hospital & Institute, Beijing 100142, China; zl20014111@163.com

**Keywords:** oral infection, *Streptococcus pyogenes*, sepsis, edentulous patients, myelodysplastic syndromes, bisphosphonates

## Abstract

Background: Infections in the oral and maxillofacial region can sometimes extend beyond the oral cavity, with serious consequences. Most oral infections are odontogenic, occurring through the root apex of the tooth or the periodontal pocket. It thus makes sense that edentulous patients have a much lower risk of oral bacterial infection. For this reason, while there are many reports on systemic infections caused by oral infections, few of these describe such infections in edentulous patients. Case presentation: We present a case of oral and maxillofacial cellulitis followed by sepsis due to *Streptococcus pyogenes* infection in an 89-year-old Japanese edentulous woman. *S. pyogenes* was detected in the wound of left maxilla and the blood sample. *S. pyogenes* has been reported to be one of the most common and influential aerobic bacteria associated with deep neck infection and subsequent systemic infection. Left maxillary sinusitis was observed, and this could be the origin of the *S. pyogenes* infection. *S. pyogenes* derived from the sinusitis and leaked to the oral cavity might have caused systemic infection through wounding of the oral mucosa. Fortunately, intensive antibiotic therapy was effective, and the patient recovered without any surgical procedures. Conclusions: We experienced a rare case of oral and maxillofacial cellulitis followed by sepsis due to a *Streptococcus pyogenes* infection in an old edentulous woman. This result indicated that, while edentulous patients are considered to have no risk of odontogenic infection, they still carry a risk of bacterial infection.

## 1. Introduction

Infection in the maxillofacial region can sometimes extend beyond the oral cavity; the abscess, cellulitis, and osteomyelitis caused by odontogenic infection sometimes lead to life-threatening systemic disease [1,2,3,4,5]. Most oral infection is odontogenic, occurring through the root apex of the tooth or the periodontal pocket [6]. It makes sense that edentulous patients have much lower risk of oral infection. Therefore, very few reports describing the risk of oral infection in edentulous patients could be found in our literature search, except for denture-related fungal infection and dental implant-related bacterial infection [7,8,9].

*Streptococcus pyogenes* (*S. pyogenes*) (Group A streptococcus (GAS)) includes human pathogens with high prevalences of infection and, in particular, is a leading cause of uncomplicated bacterial pharyngitis and tonsillitis. In addition, respiratory infections including sinusitis, otitis, and pneumonia are sometimes caused by *S. pyogenes*. Infection of *S. pyogenes* in the skin or mucosa sometimes causes cellulitis, and in rare cases, invasive *streptococci* lead to severe infections such as necrotizing fasciitis, meningitis and endocarditis. Scarlet fever and streptococcal toxic shock syndrome are systemic responses to circulating bacterial toxins [10,11]. 

Here, we demonstrate a case with systemic infection caused by *S. pyogenes* oral infection and emphasize the importance of maintaining good oral hygiene in edentulous patients. 

## 2. Case Report

An 89-year-old Japanese female was emergently admitted to the Department of Oral-Maxillofacial Surgery, Dentistry and Orthodontics at the University of Tokyo Hospital due to her high-grade fever and eyelid edema.

**Figure 1:**
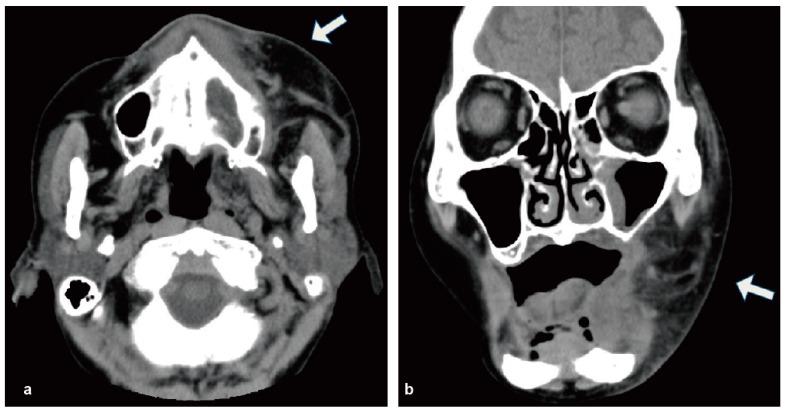
Enhanced CT revealed the soft tissue swelling widely extended to the left submandibular space without abscess formation (arrowhead). (**a**) Axial scan; (**b**) coronal scan.

**Figure 2:**
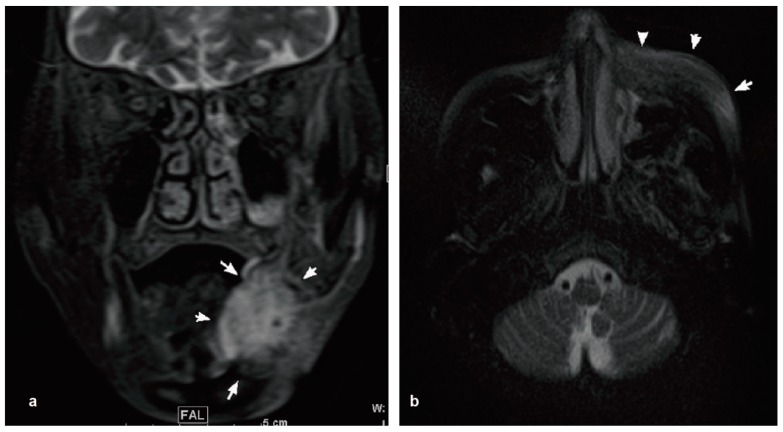
MRI indicated the swelling of the left submandibular tissue (arrowhead). (**a**) Coronal scan contrast T2; (**b**) Axial scan contrast T2.

The initial examination showed a SpO_2_ of 97%, a high body temperature (39.0 °C) and a pulse rate of 96 beats per minute. Her level of consciousness was 0 (Japan Coma Scale) and her blood pressure was 156/87 mmHg. She had no dyspnea or stridor. Her blood test showed a high white blood cell count (53.7 × 10^3^/μL, neutrophils were 82.9%). She showed anemia of chronic disease (Red Blood Cells 274 × 10^4^/μL, Hemoglobin 6.9 g/dL) and thrombopenia (Platelet 4.9 × 104/μL), C-reactive protein was elevated at 8.72 mg/dL. She had noticed swelling on the left side of her face in the days before hospitalization. She had visited a dental clinic and was told to visit an oral surgery department as soon as possible. The size of the swelling on the left side of her face gradually increased and extended between the submandibular region and the left eyelid. She had not experienced any oral or maxillofacial trauma or symptoms prior to the appearance of the swelling. The patient had no maxillary sinusitis symptoms, such as nasal discharge or obstruction. The patient had no difficulties with oral ingestion. She presented swelling, redness and a heat sensation in her left eyelid, cheek, and wing of the nose. She presented tenderness of her left face, even though it was mild. No bloodshot reaction or restriction of movement was observed in her left eye. Painless, enlarged lymph nodes with mobility were found in the cervical region. An intraoral examination found no teeth in her oral cavity and a slight scratch of the mucosa on the left maxilla. Extra oral examination revealed facial asymmetry, with obvious swelling of the left cheek. The orthopantomography confirmed that she was edentulous. Computed tomography (CT) indicated that the soft tissue swelling widely extended to the left submandibular space (Figure 1). Contrast T2-weighted magnetic resonance imaging (MRI) also indicated cellulitis from her left masticator space, the cortical layer on the inner side of the mandible body, and the submandibular space. It also showed an intense enhancing mass involving the left masticator space (Figure 2). 

This patient had been diagnosed with myelodysplastic syndromes (MDS) in 2011 and had been treated with platelet replacement therapy once a week ever since. As her other underlying diseases, she had osteoporosis and hypertension. Bisphosphonate (alendronate 35 mg) had been administrated for 5 years as a treatment for osteoporosis. For hypertension, she was taking antihypertensive drugs (calcium channel blocker and angiotensin II receptor blocker).

**Figure 3:**
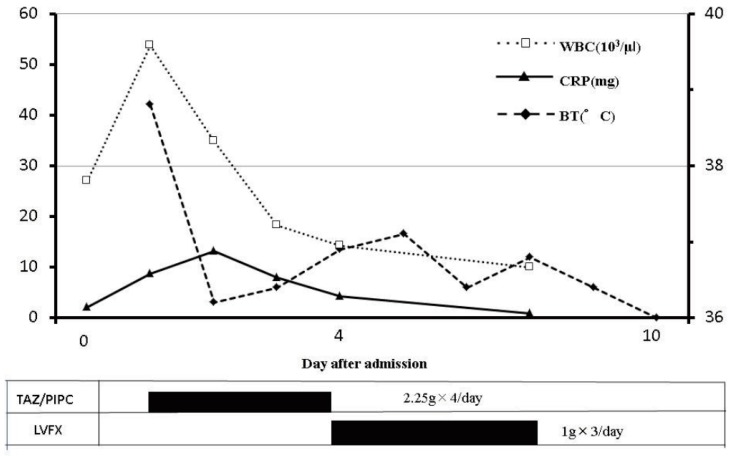
The time course of treatments and inflammatory changes. Intravenous antibiotic treatment was started on day 0. WBC, white blood cell; CRP, C-reactive protein; BT, body temperature.

An intravenous (i.v.) antibiotic treatment, Tazobactam/Piperacillin (TAZ/PIPC, 2.25 g) every 6 h, was started (Figure 3). The culture test around the oral wound showed the presence of *S. pyogenes* and *α-streptococci*. The same bacteria as in the oral wound, *S. pyogenes*, was also detected in her venous blood sample. Denture-related sinusitis by fungal infection was suspected as the differential diagnosis because she had a full set of false teeth; however, she was negative for β-d-glucan and Aspergillus antigen. After recovery of general status of this patient, the intravenous antibiotic was changed to Levofloxacin (LVFX, 0.5 g) every 12 h starting 4 days from the date of hospitalization. The sequence of antibiotic therapy was effective, and the inflammation gradually decreased. The oral wound healed and the swelling decreased. No enlargement of the cervical lymph nodes was observed. 

The wound of the left maxilla was checked at 8 days from the day of discharge, and we confirmed that the wound was clear and there were no aberrant symptoms.

## 3. Discussion

In this study, we encountered a case of sepsis caused by *S. pyogenes* in an elderly edentulous patient. There are numerous reports about systemic infections caused by odontogenic infections [12,13,14,15], but very few involving edentulous patients [7]. In such patients, denture-related fungal infection is more common, while reports of bacterial infection, especially causing systemic infection, are more limited [16]. In this case, bacteria might have entered the blood stream through mucosal injury caused by ill-fitting dentures and other means. Left maxillary sinusitis was observed in the images (Figure 1 and Figure 2) and, therefore, the sinusitis could possibly be the origin of *S. pyogenes* infection. *S. pyogenes* that has derived from sinusitis and leaked to the oral cavity could have expanded to the whole body through mucosal injury. Antiresorptive drug-related osteonecrosis of the jaw (ARONJ) is currently a big issue, and could be a focus of the systemic infection [17]. However, in this case, no obvious bone exposure was observed in the oral cavity, and the imaging studies did not suggest bone necrosis in the oral and maxillofacial region. As another possible cause of the infection, a submandibular gland infection caused by *S. pyogenes* was considered; however, no features suggesting salivary gland infection were found.

There are two major routes of infection in the oral and maxillofacial area: via the root apex, and via the deep periodontal pocket. However, edentulous patients do not have these routes. This means they have a much lower risk of oral infection. Previous reports have found that the pathogens of periodontal disease, *Actinobacillus actinomycetemcomitans* and *Porphyromonas gingivalis*, were decreased in the supragingival and soft tissue of edentulous patients [18,19].

The most common location of odontogenic infection is the mandible or maxilla into the sublingual, submandibular, or masticatory spaces, spreading into the parapharyngeal space and extended spaces [2,4,20]. Particularly, the submandibular space is more frequently involved with odontogenic infection compared to other fascial spaces [21,22,23]. In our case, an imaging study also indicated cellulitis from the left masticator space to the submandibular space.

The symptoms of odontogenic/oral infection are often unclear and tend to progress rapidly. Antibiotics therapy is the first choice of treatment for odontogenic/oral infection, but subsequent therapy must be guided by culture results, the severity of inflammation and the underlying condition. Empiric antibiotic therapy is effective at the time of a patient’s administration since it covers widely Gram-positive aerobic, Gram-negative aerobic and anaerobic pathogens [4,5]. In our case, empiric intravenous antibiotic therapy, TAZ/PIPC, 2.25 g every 6 h, was also started from the outset. *S. pyogenes* was detected from both the oral wound and blood cultures. *S. pyogenes* is reported to be one of the most common aerobic bacteria associated with deep neck infection and subsequent systemic infection [10,11].

Sepsis tends to occur particularly in patients with immunodeficiency. Previous reports have shown that patients with diabetes mellitus, chronic renal failure or other immunocompromised disease easily get systemic infectious expansion [1,2,3,4,5,12,13,14]. This patient had no diabetes mellitus or other immunocompromised disease; however, she had been diagnosed with MDS at the age of 87, and had been treated with platelet replacement therapy. MDS represents a complex spectrum of clonal hematopoietic stem cell disorders manifested by cytopenia, risk of infection, and variable risk of progression to acute myelogenous leukemia [24,25]. In this case, the patient was considered to have been in a weakened immune state. Unfortunately, the MDS had transformed into chronic myelomonocytic leukemia 2 (CMML2) at the age of 89 (one month before hospitalization in the oral surgery division). 

In this study, we experienced a case of sepsis due to a *S. pyogenes* oral infection in an elderly edentulous woman. Although edentulous people have no risk of odontogenic infection, this case demonstrates that they still carry a risk of bacterial oral infection.

## 4. Ethics Approval and Informed Consent

This research was approved by the research ethics committee of the Graduate School of Medicine and Faculty of Medicine, The University of Tokyo. Written informed consent was obtained from the patient.
